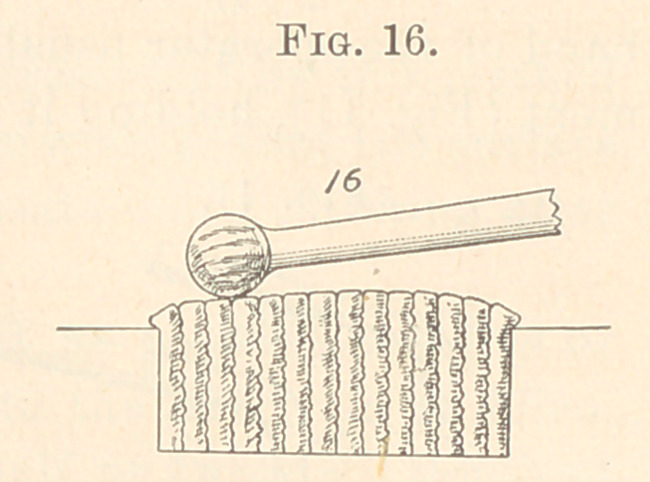# The Method of Using Soft Gold-Foil as Practised and Taught by Dr. Dunning

**Published:** 1898-04

**Authors:** Charles O. Kimball

**Affiliations:** New York


### THE METHOD OF USING SOFT GOLD-FOIL AS PRACTISED AND TAUGHT BY DR. DUNNING.[1]

BY CHARLES O. KIMBALL, M.D., NEW YORK.

Our profession stands upon the border-land between science and art, on the one hand seeking to know and on the other striving to do.

So her votaries must be clear of eye and steady of hand as well as close in observation and careful in judgment. Hence it is not amiss that we are asked to give attention for a little while to certain minute details in the use of one of the various filling-materials.

[1] Read before The New York Institute of Stomatology, February 1, 1898.

Without taking time for a prolonged discussion of the fundamental principles of filling teeth, it may be well to briefly state two.

*First.* That a good filling must exclude moisture from the tooth.

*Second.* That it must be able to resist the wear of mastication to its very edge.

And while I am not asked to argue for soft gold at all, it may be well to show why it may be wisely used, the reason being found in one word,—" adaptation." Just as we use a soft cork when we wish to stop a bottle tightly, or lead to fasten iron pipes together, so the soft gold, adapting itself readily to every minute inequality of the cavity, will perfectly exclude moisture, while it may be packed so firmly that it will resist wear as well as the enamel of the tooth itself.

The method of using soft gold-foil which I am asked to demonstrate is often called by the name of Dr. Dunning, who, taking various suggestions from Drs. Lovejoy, Rich, and others, combined them into the system which he employed till his retirement from active practice about twenty-five years ago.

The essential feature of the method consists of the packing of small pledgets of soft gold-foil against the walls of the cavity so that the structure of the filling shall be of thin plates or laminæ, extending from the bottom to the top of the cavity, usually parallel to its walls, but always perpendicular to the surface of the tooth (Fig. 1), by the means of instruments so shaped that they may

FIG. 1.

FIG. 2.

exert their pressure towards each of the walls in turn, including the bottom of the cavity, and yet without tearing the pledgets or destroying the laminæ of the filling.

For reasons which may be explained farther on, the cavities for which this method is best adapted are the small ones on the approximal surfaces of the teeth at the point of contact, and those in the fissures of the molar and bicuspid teeth, together with the cavities along the margin of the gum on the buccal side of the same teeth.

For large operations where one wall of a simple cavity has disappeared leaving a compound cavity, partly on one and partly on another face of the tooth, this method is not particularly well adapted, though in many such cases it may be used with success.

For the still larger operations, where, for instance, one entire angle of a molar tooth has been destroyed with one or more of the cusps, the method is not at all well adapted.

We are concerned with (*a*) the cavity, (*b*) the gold, (*c*) the instruments for packing it, and (*d*) the manner of using them.

(*a*) The cavity is prepared with as nearly perpendicular walls as possible, rounding out angles and carefully avoiding deep under-cuts, the ideal shape being that of a shallow pill-box (Fig. 2), following as far as possible the natural cleavage of the enamel, thus preventing the chipping of the edge under the strain of filling, and leaving the gold and tooth in the best shape for polishing.

If the decay has undermined the enamel at the edge of the cavity, it is usually best to chip the enamel back till its cleavage is over the edge of the sound dentine (Fig. 3).

FIG. 3.

The instruments by which this is done are the ordinary "hatchets" and "hoes," but in order to get a perpendicular side and a clean square edge we take an ordinary hatchet and grind its front face (Fig. 4) so that it cuts with its edges; the hoe is treated on

FIG. 5. FIG. 4.

the inner face (Fig. 5) in the same way. By these two used alternately we can follow round the cavity (Fig. 6).

Of course, there are other instruments used, but these two used in this way are particularly helpful in making the walls of approximal cavities perpendicular to the surface.

Fig. 6.

Fig 7.

Approximal cavities require for their treatment by this method separation of the teeth by wedges or otherwise, so that the surface of the filling may be reached for condensation and finishing after it is inserted.

(*b*) The gold, which should be as soft as possible, is rolled or folded in pellets, of a size proportioned to the cavity to be filled, their diameter being about once and a half or twice the depth of the cavity.

Their density is directly proportioned to the strength of the walls of the cavity, for frail walls softer pieces being used.

(*c*) The instruments are shaped like a square truncated pyramid bent at various angles and of various sizes, the most useful being those nearly at right angles with the handle (Fig. 7), and those about forty-five degrees with the handle (Fig. 8). The size for each cavity is such that the blade reaches to the bottom of the cavity without the handle touching the edge, but without having much to spare (Fig. 9). They should be tempered so as to neither bend nor break,

Fig. 8.

Fig. 9.

though it is better to have them break than to bend. The faces should be smooth and polished, the angles very sharp, and the little terminal face or facet exactly square with the point and sharp on every angle. For condensing, the same instruments are often used, but more frequently smaller ones are required (Fig. 10), somewhat more abrupt in their taper, shorter in length, and smaller in the terminal facet. There should be also a very small round plugging bur cut fine, with round and roughened handle, tempered like a plugger to spring to its point.

FIG. 10.

(*d*) The manner of packing is to press a pellet of gold of suitable size firmly against the wall of the cavity, most distant from the hand of the operator usually, with the side or end face of the instrument (Fig. 11), holding it in place by a second instrument held in

FIG. 11.

FIG. 12.

the left hand, or by an adroit catching of the pellet in the wall of the cavity, and then packing it firmly against the wall by repeated thrusts of the instrument, making the pressure always towards the walls and perpendicular to them, while the blade of the instrument is parallel to them. This tends to press the gold into a flattened mass tight against the side of the cavity, reaching to the top in an unbroken sheet (Fig. 12), bulging out a little above the edge, but solid from bottom to top. To this another piece is added, changing slightly the direction of the pressure so that it is still perpendicular to the wall but at another point (Fig. 13), but packing with great

FIG. 13.

FIG. 14.

care, not leaving any piece until it is packed as hard as possible, or as hard as may be best. This process is repeated gradually, also reducing the size of the pellets as the cavity remaining unfilled becomes smaller, until it is reduced in size to a mere pit near the centre of the original cavity, or rather nearer one end or side; this is then filled with smaller pellets, using the flattened point of the instrument or a smaller one (Fig. 14). Now, if the process has been thoroughly done the gold should be quite solidly packed, of a fairly even texture, no softer at the bottom than the top, but still too soft to leave a good or safe surface. Next comes the

*Condensing.*—The filling is pressed by the flattened point of the instrument or by one having a smaller truncation (Fig. 15), working the gold solidly into the cavity. It is then rolled with a plugging bur, which is a round-handled, fine-cut bur of very small size, but tempered like a plugger (Fig. 16), and burnished with a small ball-shaped burnisher to condense the surface, and finally is finished off with knife, file, corundum, pumice-stone, and rotten-stone till the surface of the filling and the tooth are continuous.

FIG. 15.

FIG. 16.

Such a filling is well adapted for a grinding surface because the gold will never flake off; it is like a pavement of thin gold blocks laid on edge, each holding the other in place. It is also good for a small approximal cavity, because the instrument works equally well in all directions, and so will pack towards or from the operator's hand.

## Figures and Tables

**Fig. 1. f1:**
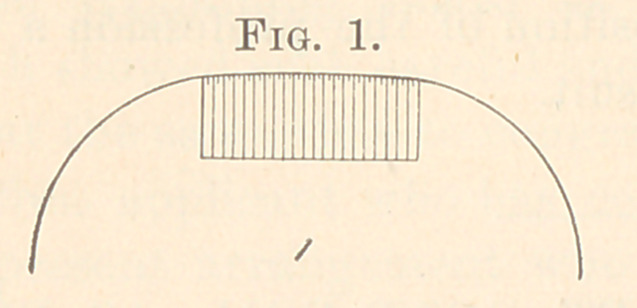


**Fig. 2. f2:**
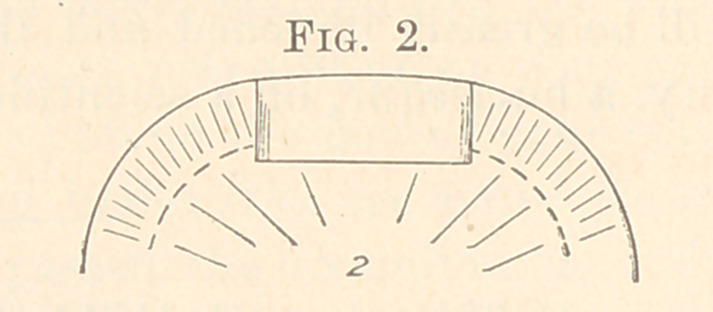


**Fig. 3. f3:**
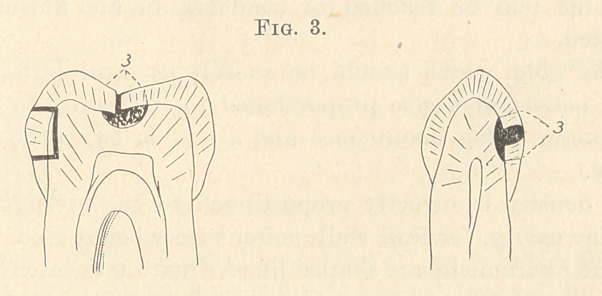


**Fig. 5. Fig. 4. f4:**
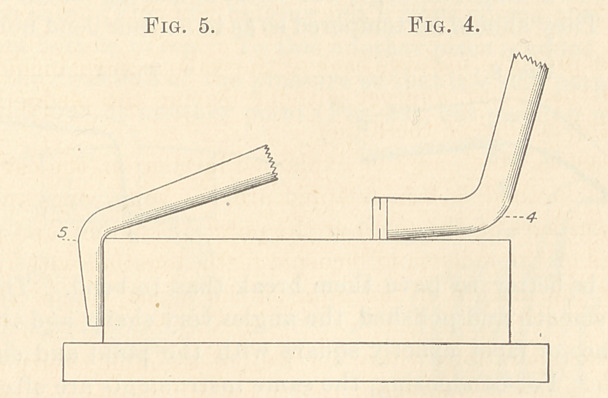


**Fig. 6. f5:**
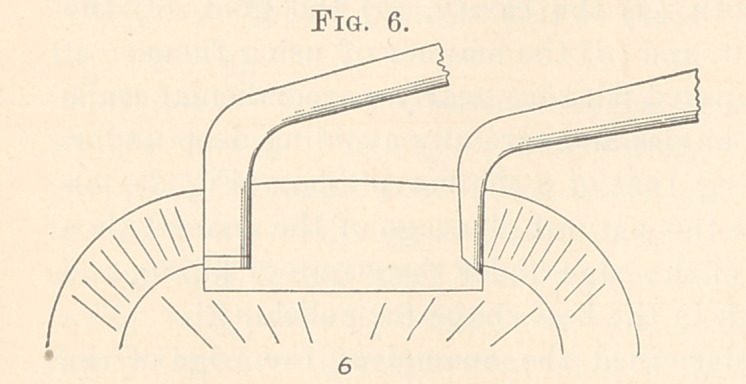


**Fig. 7. f6:**
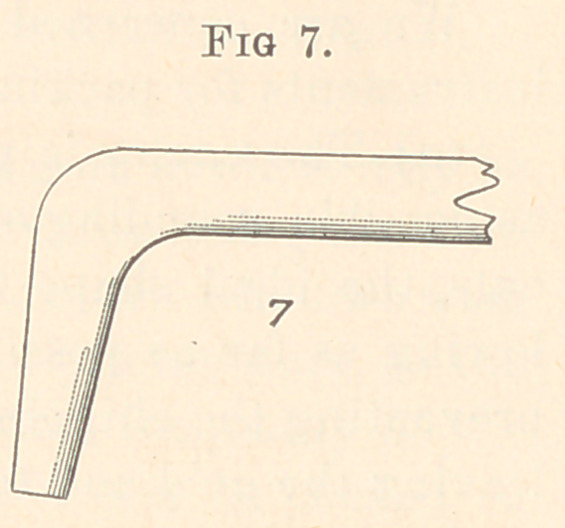


**Fig. 8. f7:**
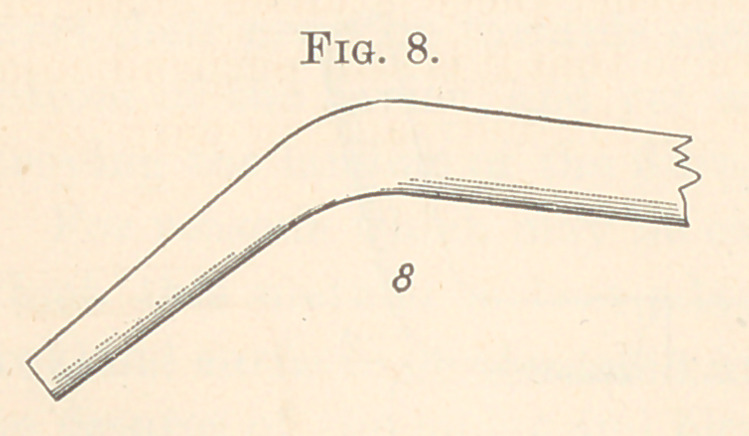


**Fig. 9. f8:**
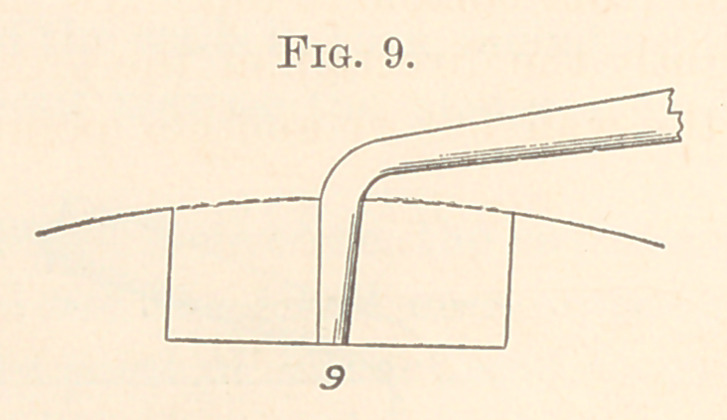


**Fig. 10. f9:**
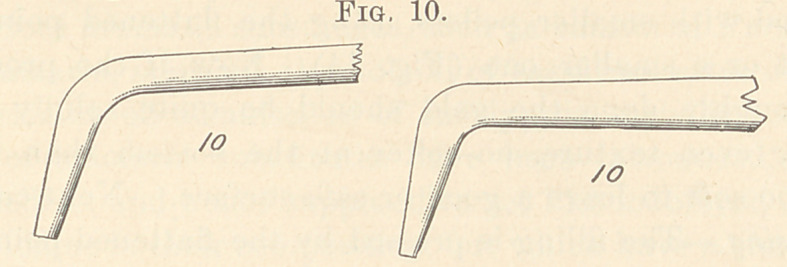


**Fig. 11. f10:**
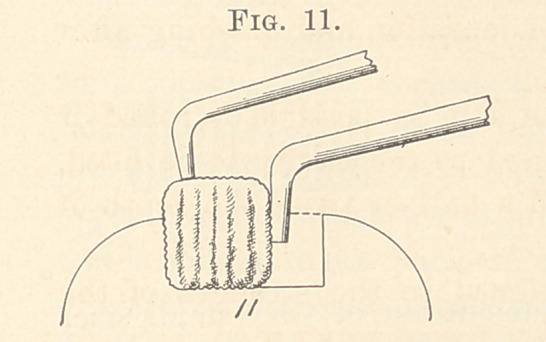


**Fig. 12. f11:**
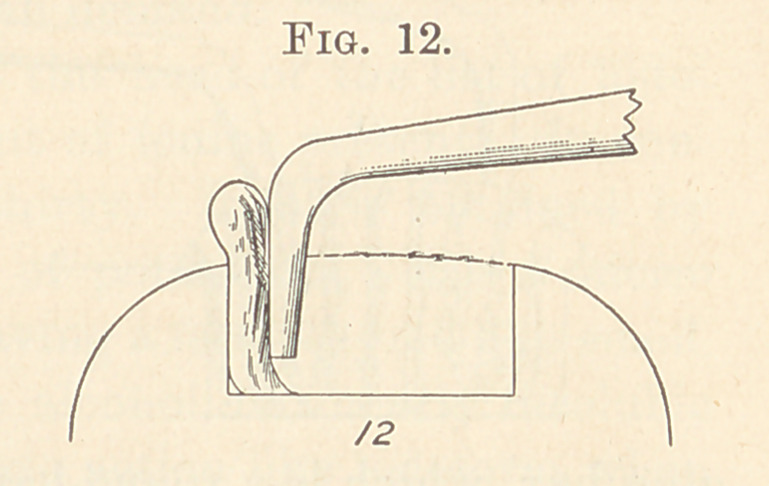


**Fig. 13. f12:**
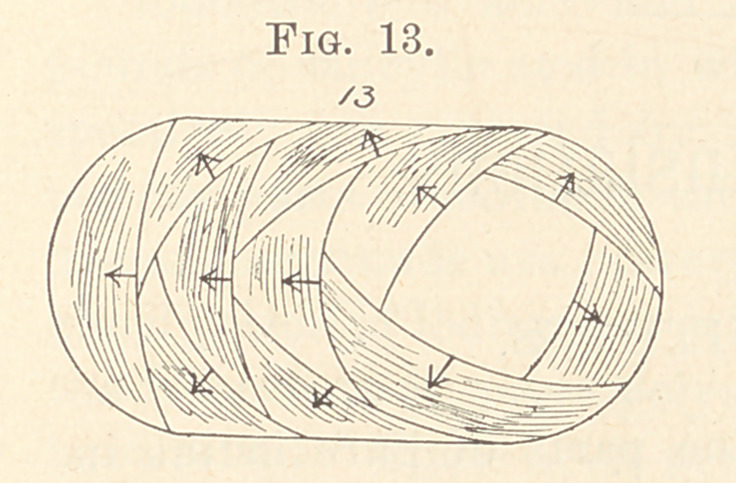


**Fig. 14. f13:**
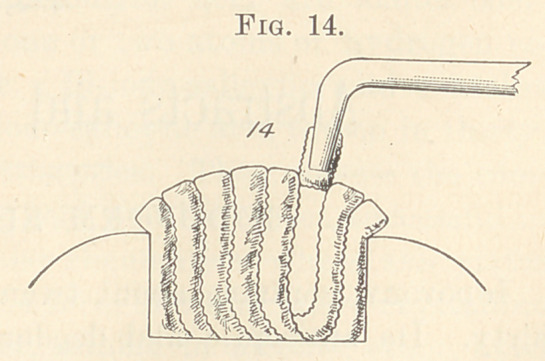


**Fig. 15. f14:**
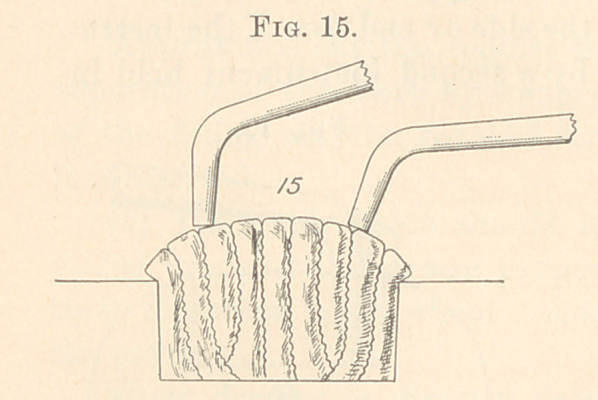


**Fig. 16. f15:**